# Doctoral students’ perceived working environment, obstacles and opportunities at a Swedish medical faculty: a qualitative study

**DOI:** 10.1186/s12909-019-1684-x

**Published:** 2019-07-08

**Authors:** Lena Ljungkrona Falk, Hanna Augustin, Kjell Torén, Maria Magnusson

**Affiliations:** 1 Section of Occupational and Environmental Medicine, Institute of Medicine, Sahlgrenska Academy, University of Gothenburg, Gothenburg, Sweden; 2 Primary Healthcare, Närhälsan, Västra Götaland, Sweden; 3 Department of internal medicine and clinical nutrition, Institute of medicine, Sahlgrenska academy, University of Gothenburg, Gothenburg, Sweden; 4 Angered Hospital, Gothenburg, Sweden

**Keywords:** Doctoral students, Research, Supervisors, Working environment

## Abstract

**Background:**

Investment in research is high on the agenda of many countries in recognition of the fact that research is important for the development of society. Doctoral students have a vital role and represent a substantial part of this investment. It is therefore imperative to reduce the risk of students dropping out from doctoral studies. The aim of this qualitative study was to gain deeper insight into the working conditions of, and obstacles and opportunities for, doctoral students at an institute of medicine in Sweden.

**Methods:**

Semi-structured interviews were conducted in 2013 with 17 doctoral students—of varying genders, professions and fields of research—from the Institute of Medicine, Sahlgrenska Academy, at the University of Gothenburg, Sweden. The recorded interviews were transcribed and analysed using systematic text condensation.

**Results:**

Four categories emerged from the data. They were: Safety, Frustrating Structures, Others - not me, and the future. They included positive as well as negative perceptions. Among the positive perceptions were recognition of the importance of the supervisor, as well as secure conditions, and personal development. Frustrating structures in the academic culture, stress and differences in career building constituted the negative points.

**Conclusions:**

Our findings suggest that there is a need for structures within the university that support doctoral students who feel they are not receiving the assistance they need, who believe they have unreasonable working conditions, or who may need to change supervisors in order to complete their graduate research studies. Our study also highlights the fact that supervisors have a major influence on the work environment of doctoral students, and that the general and academic perception of the research area likewise has an effect on the successful completion of the research project and dissertation. Providing leadership training for supervisors could be an important measure that may help improve conditions for the doctoral students they supervise.

**Electronic supplementary material:**

The online version of this article (10.1186/s12909-019-1684-x) contains supplementary material, which is available to authorized users.

## Background

Investment in research is high on the agenda of many countries in recognition of the fact that research is important for the development of society. Among Organization for Economic Co-operation and Development (OECD) member countries, Sweden is ranked as one of the leading investors in research and development in relation to the country’s gross domestic product [1, 2].

During the last decade, about 3000 students enroll yearly for doctoral education in Sweden [3]. About one third of them are within the research field of medicine and health [3]. Doctoral education is an economical investment for society and an investment of time and effort for the individual student. In Sweden, a doctoral education include 4 years full time studies. Institutional or teaching tasks may prolong the time, commonly with another year. The majority of these students are full time employed by the university. There are also doctoral students who are employed by e.g. the university hospital, should be able to use 50% of their employment hours for doctoral studies and 50% for clinical work e.g. as medical doctors, psychologists, physiotherapists, dietitians, or other medical professions. This means that the time needed to complete a doctoral education in Sweden varies from one individual to another. Over the last 20 years about 40–49% have obtained their doctoral exam within 5 years after enrolment [3]. Among those enrolling in 2009 within the fields of medicine and health, about 80% had obtained their doctoral exam within 8 years [3]. These figures indicate that more than 20% of candidates experience delays and interruption of their doctoral education. A better understanding of the nature and extent of the underlying causes of such delays is vital, if the system is to be improved.

So far, studies have shown that timely completion of a doctoral degree requires a variety of factors viz.*,* social support, and quality of supervision, work environment and access to the research culture [4–7]. A crucial common denominator seems to be recognition of the doctoral student at different levels, including the supervisor, colleagues and the research culture. Having access into the research culture avoids isolation and a feeling of not being integrated into the research community at the university [7]. The supervisor has a pivotal role for this, and for being supportive on project issues as well as other issues important for the doctoral student [8, 9]. According to Manathunga et al., experienced supervisors meet their doctoral students on a regular basis so that they may identify warning signs of issues that need to be dealt with to avoid delays or even attrition [4]. Nevertheless, many doctoral students find themselves in surroundings riddled with psychosocial issues and work environment dilemmas [1, 10–12].

In Sweden, the Department for Higher Education has carried out two questionnaire surveys with the purpose of identifying factors associated with the interruption of doctoral studies [10]. The major reason identified was social factors, including e.g. dislike being a doctoral student and difficulty combining doctoral studies with family needs. Examples of other social factors mentioned in the relevant literature that were associated with interruption were that female doctoral students expressed a feeling of gender-related discrimination [13] or described experience of discrimination or sexual harassment [14–16]. This trend was corroborated also by the unpublished results of a questionnaire study among doctoral students at the University of Gothenburg, conducted in 2012 (*n* = 114; 86 women, 55 men), where female doctoral students had expressed gender-related discrimination, as compared with their male counterparts. The same study found that doctoral students perceived their work as demanding, but felt compensated by the flexibility in the working process (Kjell Torén, personal communication).

Therefore this research group chose to evaluate the results of the quantitative survey by performing complementary qualitative individual interviews with doctoral students both working full time and part time with their doctoral studies.

Thus the aim of this qualitative study was to gain deeper insight into doctoral students’ working conditions at the Institute of Medicine, the Sahlgrenska Academy, University of Gothenburg. We set out to explore both obstacles to the successful completion of their studies, and opportunities they are offered. This knowledge may serve as the basis for the development of initiatives that foster better working conditions.

## Method

### Recruitment of participants

Participants were recruited from among doctoral students at the Institute of Medicine, Sahlgrenska Academy and University of Gothenburg (IoMUGOT), between March and October 2013. The Committee for Post-Graduate Studies at the Institute of Medicine, has the responsibility of evaluating and improving the working conditions of doctoral students, and the Committee assisted by providing a list of current doctoral students. The Committee, consisting of researchers from IoMUGOT, has no personal interest in the research projects of the doctoral students [13]. When selecting students the research group aimed for variety concerning gender, profession, form of employment and field of research. By these criteria 29 doctoral students were selected from the Committee list.

An email was sent separately to each of them. The email included information about the study and an invitation to participate. Further contact via telephone or email was taken up with those who expressed an interest in participating. Additional recruitment was conducted by snowball sampling, i.e. interviewees were asked to suggest potential participants. The snowball sampling had two aims: to ensure that we would collect as rich and interesting a dataset as possible, and to increase the number of participants [17]. Twelve of the first 29 invited students participated. Following the suggestion of these 12 participants, five more participated, so in total, 17 interviews were conducted.

The anonymity of all participants was guaranteed. The recruitment process and the study protocol were approved by the Regional Ethical Review Board in Gothenburg (diary number: 471–11; T043–13).

### The interviews

An interview was booked when the participant had given informed consent either orally or by email. The interviews were conducted by the first author (Ljungkrona-Falk L.) who has many years of experience as a reg. dietician. She has published two scientific papers, had no connections to the institution and was employed as a research assistant. The interviews took place at a location chosen by the participant. All this was done to ensure as little impact as possible on the participants [18].

Before an interview began, the interviewer explained that the participant could withdraw their participation in the study at any time and request that their data be deleted, without giving a reason. The participants then signed an informed consent form. The interviewer also stressed the aspect of anonymity.

The questions in the interview guide consisted mainly of open-ended questions. The opening question was: *“How are things in your life and around you right now?”* followed by four key questions about positive and/or negative experiences from their research environment. The interview closed with the question, *“Is there anything you want to add?”* [18, 19]. After the first interview, a question was added allowing the participant the opportunity to express any criticism regarding their work situation. The modified interview guide was subsequently used for the remaining interviews (see Additional file 1 ; Interview guide, COREQ gudielines [18]).

Interviews generally lasted 60–120 min. Brief notes were taken during the sessions. A great effort was made to create an open, friendly atmosphere so that the participants would feel comfortable in the interview situation [18, 19]. The interviews were digitally recorded and the recordings were personally delivered to a secretary by the first author. During the transcription of the interviews, all names of persons, cities, workplaces, institutes and other information that could reveal participants’ identity were anonymized. The audio recordings and other documentation were stored on the server in the university’s safety information management system. Access to the material was by secure login only available to the researcher.

### Analysis

Malterud’s method of systematic text condensation was applied as the method of analysis [19–23]. In accordance with this, the first author read and listened to each interview repeatedly. Meaningful units of analysis were identified and coded. The transcripts were read and recoded multiple times in parallel with conducting new interviews and adding new meaningful units of analysis. The codes emerged as primary themes and were divided into sub-groups, describing variations within each code. The last author read each interview, and listened to and coded parts of the material. The codes were subsequently compared. Where discrepancies were detected, these were discussed until consensus was reached. Due to the continuous nature of the analysis, the interview material was defined as saturated when no new information regarding the aim of the study was added.

After the analysis was completed, the first author read through the interviews to scrutinize the materials for anything contradicting the results. Preliminary results were presented to representatives of the university’s student union for doctoral students, at an open discussion meeting with 12 participants, who confirmed that the findings mirrored the reality of the students.

## Results

**Fig. 1:**
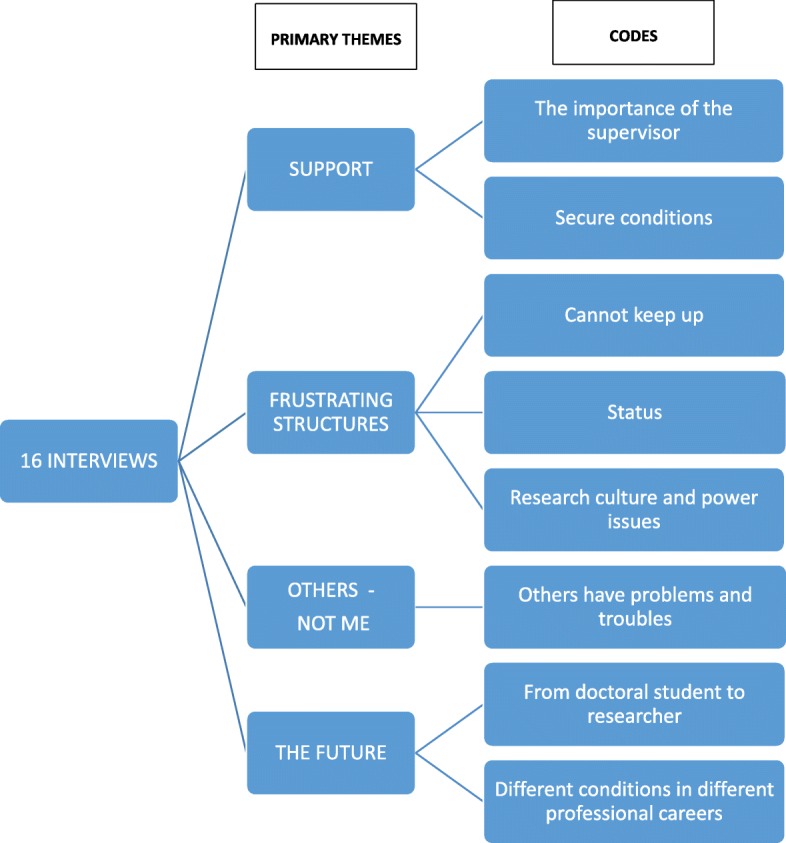
The analysis process of systematic text condensation categorizing the interview materials into primary themes and codes

To ensure anonymity, no professional title other than “physician” was disclosed in the analysis, and professions were therefore categorized into two groups, “physicians” and “others”. The participants were a mix of full-time doctoral students (*n* = 5) and students who were working clinically in parallel to their doctoral studies (*n* = 12). Results are based on 16 interviews, since one participant decided to withdraw from the study. Of the 16 participants, eleven were women and five were men. For headings of themes (codes) and sub-groups (variations of codes), see Fig. 1. In the following, quotations are presented under the respective headings, with participants denoted by numbers corresponding to the order in which they were interviewed.

### Support

#### The importance of the supervisor

Eleven participants were very satisfied with their supervisor and said that they were given ample support. Some stated that it was more important to have a good, well-funded supervisor than to have an interesting research topic. Important traits of a good supervisor were said to be: easy to access, supportive, a good listener and showing an understanding of students’ life situations. Two participants emphasized that creating a good work atmosphere and building strong relationships is a mutual responsibility between the doctoral student and their supervisor.

***I think my current supervisor is unusually good at listening and is a very supportive person, despite being extremely busy. (I:1)***

Dissatisfaction with the supervisor was based on different kinds of problems between the supervisor and the student (e.g. personal problems, disagreements, misunderstandings). There were also examples of inability of the supervisor to handle conflict with other members of the research group, and failure to give feedback, which could lead to loss of research time. The lack of feedback made some students (*n* = 5) express uncertainty about whether they had made the right decisions. Two participants described themselves as incompetent and blamed themselves for lack of progress in their work.

The feeling of dependency on the supervisor was described as very strong and as existing at different levels. This was exemplified by a situation in which a key person in a research group moved away and several research projects were discontinued, which resulted in some doctoral students dropping out of their studies. The research process and the doctoral student’s position become very vulnerable when a certain research project is dependent on individual researchers and there is no backup plan in place.

Nine participants expressed their reluctance to criticize their supervisor because of worry that this might lead to bad references or because they thought that the supervisor had contacts crucial for their future.

***If you don’t get good guidance and support from your supervisor, it becomes unsustainable because you get no direction (I:1) It’s a difficult situation we***’***re stuck in. (1:17)***

#### Secure conditions

Several (*n* = 9) of the participants said they believed that full-time employed doctoral students often had better work conditions compared with those who worked clinically alongside their doctoral studies.

***I have been a full-time employee doctoral student from the start, so in that way it has been great conditions also in terms of pay (1: 2)***

They also saw a big difference in how the researchers’ salary was funded as well as how their work situation was organized. Some (*n* = 3) worked every other week on their research and had the same schedule as their supervisor, which vastly facilitated the process. Regardless of the manner in which the participants were employed, most of them (*n* = 15) felt that it was sometimes inevitable to have to conduct research during evenings or on the weekend, although some of their supervisors maintained that research should be conducted during paid working hours only. Four participants stated that they felt very secure in their role as researcher and that there were good contracts and backup plans in place. They had a trusting relationship and communication with their supervisors, who willingly and in an encouraged manner, discussed research tasks and work in the future which promoted a secure working milieu.

To be a research clinician was considered by many (*n* = 10) as very positive, since the work situation was not dependent solely on the research.

Some participants (*n* = 3) viewed research as something that is done out of passion. Those who were physicians felt safer than other professions because they could always go back to their clinical work after the public defence of their dissertations, and some could continue to combine clinical and research work.

***I can tell you that they are providing for me very well. They are very keen on having me stay on. (I:12)***

### Frustrating structures

#### Cannot keep up

Twelve of the participants described stressful conditions arising from having to conduct their research during their spare time and on weekends. Clinicians who were interviewed said that many do research during their standby time. Part-time doctoral students felt that combining their regular work with research was stressful. Some also had a bad working milieu when working on their thesis (e.g. four people shared an office meant for one). These participants stated that their thoughts and concentration were frequently interrupted. For five clinicians, research was a way to cope and manage feelings of stress and pressure from many patients.

***There is always some stressor that worries away inside you. I am expected not only to deal with the regular stuff, but to manage all the extra stuff as well. (I:15)***

#### Status

Most of the participants (*n* = 13) conveyed that they were part of a hierarchical system with built-in structures, which they perceived as having nothing to do with research. Some mentioned that there was an unstated requirement that they should participate in certain activities simply because they were doctoral students. One participant gave an example of doctoral students whose time and low hierarchical positions were taken advantage of when they were assigned to manage laboratory mice that were to be used in other people’s projects. There was also mention of problems in getting results published if the doctoral student came from a non-European country (with the exception of the US). One participant who was part of a research group felt alienated and was left out of e-mail conversations among the other members in the group. Another doctoral student did not receive a response to his manuscript for a long time, and when they did, the response was very brief. The student believed that the supervisor had not responded to the manuscript because of its poor quality. This student also blamed themselves when the chief supervisor and the co-supervisor had a conflict, and did not dare to talk about it to anyone for fear of not being believed.

***You are so afraid that people won’t believe you, and that this person is going to speak badly of you behind your back and that everyone else is going to believe this person instead of having faith in a doctoral student who hasn’t yet produced anything. (I:17)***

None of the participants in our study felt any gender-related discrimination. One participant stated that the courage to speak up was a question of personality and not linked to gender. Some (*n* = 4) sensed that their colleagues in the clinic thought that they “took time off” and “relaxed” while conducting research. Another worry was the uncertain employment situation and the lack of a safety net. For example, if one were to become ill while doing research on grants, there was no health insurance available.

#### Research culture and power issues

Twelve of the participants stated that though the environment and culture at the university were fantastic there were several unstated requirements. One was the expectation that they work more than was stated in the employment contract. One doctoral student who wanted to reduce the working hours while on parental leave was advised by their supervisor to skip out on snack time and eat lunch in front of the computer instead. As described by seven participants, another example of possibly irregular behaviour within the university culture was that some more senior researchers were listed as authors in papers to which they had not contributed. Supervisors had stated that students simply had to accept this reality.

Several of the participants (*n* = 10) described a fear of getting into a conflict with their supervisor and research group. Five participants also gave the opinion that though the culture might be impartial, the system did not always work perfectly.

***The greatest injustice is the total dependence on the supervisor, and because of these interpersonal relationships, winding up in a bad situation can be a particularly tough nut to crack. In a “regular work environment” I would have an entire union organization to back me and there are systems and means of bringing a difficult situation to light. But as a doctoral student, making a complaint or questioning anything could jeopardize my entire project. There is always someone else waiting in the wings who is willing to take up the project, so one is very vulnerable as a doctoral student. (I:14)***

Three participants who were employed as doctoral students and had not made their research plan themselves found that much of the allotted time was spent making the project into their own research project. There was ambivalence and frustration as the research came to a halt and they lost time as well as money. Examples included difficulties in recruiting subjects for studies or in getting their articles published. One participant who had received a large grant for her research felt a great responsibility and worried that the research would not make a big enough contribution back to society.

### Others – not me

#### Others have problems and troubles

Several participants (*n* = 12) said that they were doing fine, but that many others had problems. They were constantly noticing issues in their surroundings without being personally affected. Many stated (*n* = 14) that they did not have the courage to express criticism because they were uncertain where to turn or what the consequences might be.

One participant noted that others had problems with supervisors disfavouring them on the basis of personal chemistry, apparently a problem that occurred higher up in the organization. There were stories of supervisors using master suppression techniques to get results from their doctoral students; also there were stories of others who had to conduct research almost entirely in their spare time, evenings and weekends. The informants who tell this said that they: “heard this from others or about others”.

***I’ve heard stories of supervisors who are … how do I say this … sociopaths who are prone to pressure their subordinates to get results. (I:14)***

These participants typically regarded their own situation as satisfactory and their supervisors as supportive, but stated that “others” lacked this kind of support. “Others” also had no funding other than grant money, and received no support for parental leave or illness.

***Even if I haven’t been significantly affected personally, there have been supervisors known to let personal chemistry decide whether to favour or disfavour someone. Unfortunately, bias can also occur. I have seen it, even if I haven’t experienced it myself. And surprisingly, it primarily occurs with the people in high-up positions. (I:10)***

### The future

#### From doctoral student to researcher

Doctoral students in this study expressed happiness and said they had found passion in learning and had grown into the role of researcher.

***I think that you grow into the role as a researcher and it’s a big personal change. You change your vocabulary, your posture – and you gain a completely different outlook. (I:2)***

***If I think about what I knew before … Wow, you just learn so much. (I:12)***

Most of the participants (*n* = 14) cited the flexibility of the work situation and the ability to control their own time as important components of coping. Some (*n* = 3) considered research as a way of seeing their work in a different light and of gaining insight into various guidelines and methods of treatment. Some of the part-time doctoral students, both male and female, felt questioned in their regular workplaces and said there was a lack of understanding for the work of a researcher.

Getting into a research group was highlighted as particularly significant because, as participants recognized, personal relationships affect the work situation, as does the researcher’s research ability.

Participants also felt that the time prior to thesis defence was highly stressful and intense due to time constraints when finalizing everything included in the thesis.

#### Different conditions in different professional careers

It was more common to feel anxiety about the future, after the dissertation, among doctoral students who were not physicians. Students who were physicians were aware that their future was more secure compared with many other professional fields. Often there was no clear-cut plan for other professionals. It was deemed, in some professional fields, that having a doctoral degree might be a burden, rather than an advantage, because having a degree can make a person a more expensive employee. The interviewed physicians had also reflected on this and some raised the point spontaneously during the interview. A common view among all participants was that one simply did not become unemployed as a physician. Several participants felt stressed when others asked what they were going to do after the dissertation. The non-physicians were unenthusiastic about continually having to apply for grants to secure their future incomes, especially if they had families to provide for. At the same time, there was frustration over not getting to utilize their knowledge as researchers in a meaningful way.

***Those who are pure researchers are probably more vulnerable than clinical workers, since they often have some form of employment safety, and reassurance that they can get jobs once they have defended their theses. This is also true when it comes to recommendations and good references. There’s a pretty complex network surrounding this and it’s very uncertain when you have to go out and look for a job. (I:1)***

## Discussion

The main findings in our study were the contrasting experiences between clinical working part time doctoral students, who worked clinically in parallel, and full-time doctoral students. There were also differences in working conditions between physicians and other professionals, related both to economic conditions during period as doctoral student and options for post-dissertation career building. None of the physicians worried about future employment. They would always have the opportunity to work in the clinic if they did not continue with research.

Our findings further confirm that the supervisor is crucial for doctoral students’ success, regardless of the students’ profession. The skills to supervise and give psychosocial support are of great value. The influence and importance of the supervisor and their support has been shown in other studies, both within the Swedish context and from other parts of the world [4, 5, 24–28]. It is interesting that our study, which is limited to one academic centre in Sweden, shares experiences with other settings. For example, a recent publication from Iran [29] reports several similar experiences despite a completely different context. The authors argue that it is of great importance to have a good relationship with one’s supervisor. As in our study, the doctoral students in that study experienced stress over different aspects of funding their research, and also they had great stress in their daily life and concerns about post-dissertation employment. Other studies in this field also show that pressure and attrition are high among PhD doctoral students [24, 25]. Frischer & Larsson (2000) show that a paucity of leadership from supervisors, sometimes referred to as “laissez-faire leadership” or, as they term it, “a leader nominated but abdicated” can result in unfinished doctoral studies [8]. Studies from other contexts suggest that supervisors should undergo training so that they can recognize early warning signs of extreme stress among their research students and prevent potential problems. This may help ensure timely completion of the research and students’ dissertations [4, 29, 30].

Doctoral students with knowledgeable supervisors of high academic standard and good financial capability seem to have greater enthusiasm and be more resilient [28, 30–32]. Given the vital role of the supervisor in the research process, institutions should be encouraged to give more support and training to their supervisors. In situations where the supervisor has obvious problems, the university needs better back-up plans. Our results imply that the supervising role should not be based on academic position and knowledge in the specific field alone, but should also consider other qualifications. Supervisors are vital in securing the future of the research, setting the path for continuous research of high quality, and but also improving the working environment of doctoral students. Continuous education of supervisors in coaching and supervision is needed to secure and improve the situation for many doctoral students. This was also one of the reasons the Association for the Study of Medical Education (ASME) in the UK arranged an education research consortium to provide support for supervisors [9].

The conditions included hard work, which most of the doctoral students in our study declared was largely compensated for by the learning processes and the positive personal development that were entailed.

Working conditions of and employment arrangements for doctoral students differ between countries. In Sweden, medical doctoral students can proceed their doctoral studies full-time or part time (at least at 50%) during their doctoral education. However, expectations on when the work should be done differed within the institution regardless of the form of employment. Two of the students had supervisors who made it clear to them that they should not do research in their standby or spare time, while others presupposed that research was carried out after work hours. The exact arrangement seemed to depend more on the supervisor and the culture within the group than on formal rules in different institutions.

In our study, women did not seem to have more problematic working conditions than men, although such differences has been reported in other studies [7, 13, 24]. The results from our survey questionnaire could reflect the fact that the single most common profession represented was physician (in both male and female participants). Participants from other professions (male and female) expressed greater difficulties, both in securing funding through grants and in securing a position in the academic world. They also seemed less confident regarding their future careers. Ultimately what most seemed to determine the self-perceived work capacity was the supervisor and the stress load.

One might have expected that a number of doctoral students with negative experiences would agree to participate in our study in order to express their criticism and suggest improvements. However, this does not seem to have been the case. The participants were careful when speaking up, but several stated that “others” were experiencing problems. An alternative interpretation could be that it is much safer to say that other people are having the problems that you are experiencing, thus taking the opportunity to make your voice heard but hiding behind “others, not me”. This was unexpected since great efforts were made to provide safe environments during interviews and to prevent tracing of outcomes. However, the institution/supervisor may appear so extremely powerful that it would be dangerous to reveal criticism even under such conditions.

It is interesting to note that the participant who dropped out and requested that the interview be deleted was the one who faced the hardest conditions (inclusion of this information has been approved by the participant in question). This says a lot about the effort of being a doctoral student, but it also demonstrates the fear of perhaps not receiving good future references. It is also possible that some doctoral students feared that participation in this study would be perceived as criticism of their supervisor or institution.

This study was limited to one large institution, in one country, i.e. with specific social and economic conditions. Also the snowballing approach may induce bias. Since only three of the respondents were recruited this way we estimate the risk of bias to be low. However, some of the findings and conclusions may be transferable to, or illuminate, similar situations, questions and problems in other, similar contexts. The study’s credibility, or truth value [23], was confirmed by two members of the Doctoral Students’ Council at the University of Gothenburg who explicitly recognized the significance of results and quotations. By asking the Committee for Post-Graduate Studies at IoMUGOT to suggest potential participants we achieved a variety in our sample and got in contact with participants with substantial experience.

## Conclusions

Our findings suggest that there is a need for structures within the university that support doctoral students who feel they are not receiving the assistance they need, who believe they have unreasonable working conditions, or who may need to change supervisors in order to complete their graduate research studies. Our study also highlights the fact that supervisors have a major influence on the work environment of doctoral students, and that the general and academic perception of the research area likewise has an effect on the completion of the research project and dissertation. Providing leadership training for supervisors could be an important measure that may help improve conditions for the doctoral students.

## Additional file

Additional file 1: The Interview guide. COREQ guidelines [18]. (PDF 147 kb)

## Data Availability

Data cannot be made freely available as they are subject to secrecy in accordance with the Swedish Public Access to Information and Secrecy Act [Offentlighets- och sekretess-lagen, OSL, 2009:400], but can be made available to researchers upon request (subject to a review of secrecy). Requests for data (de-identified) should be made to Lena Ljungkrona Falk, e-mail: lena.ljungkrona-falk@vgregion.se. Requests can also be addressed to the Head of the Department of Internal Medicine and Clinical Nutrition, University of Gothenburg.

## Abbreviations

ASME: Association for the Study of Medical Education
IoMUGOT: Institute of Medicine, Sahlgrenska Academy and University of Gothenburg
OECD: Organization for Economic Co-operation and Development
OSL: (Offentlighets- och sekretesslagen) Publicity and secrecy law (2009: 400)

## References

[1] Ehn Knobblock I, Bender G. Hur mår doktoranden? [How does the doctoral student feel? Stockholm: Federation of Labour Unions; 2012.https://st.org/sites/default/files/attachment/hur_mar_doktoranden_juni_2012.pdf. Accessed 28 June 2019.

[2] OECD Publications and Information Centre. Postgraduate education in the 1980s. Washington: Paris Organisation for Economic Co-operation and Development; 1987. https://eric.ed.gov/?id=ED286415. Accessed 28 June 2019. ISBN: 92-64-12980-4.

[3] Universitet och högskolor. Doktorander och examina på forskarnivå 2016. [Postgraduate students and degrees at third cycle 2017]. Statistiska Centralbyrån, Sverige [Statistics Sweden]. https://www.scb.se/contentassets/632f377ba0404d3e93641b7a48564db9/uf0204_2016a01_sm_uf21sm1701.pdf. Accessed 28 June 2019.

[4] Manathunga C (2005). Early warning signs in postgraduate research education: a different approach to ensuring timley completions. Teach High Educ.

[5] Delamont S, Atkinson P, Parry O (1997). Critical mass and doctoral research: reflections on the Harris report. Stud High Educ.

[6] Lovitts BE (2001). Leaving the ivory tower: the causes and consequences of departure from doctoral study.

[7] Morgan CK, Tam M (1999). Unravelling the complexities of distance education student attrition. Dist Educ.

[8] Frischer J, Larsson K (2000). Laissez-faire in research education – an inquiry into a Swedish doctoral program. High Educ Pol.

[9] Pugsley L (2008). Expectation and experience: dissonances between novice an expert perceptions in medical education research. Med Educ.

[10] Rapport 2012:1 R. Orsaker till att doktorander lämnar forskarutbildningen utan examen – en uppföljning av nybörjarna på forskarnivå läsåren 1999/2000 och 2000/01. [Reasons why doctoral students leave the postgraduate program without a degree - a follow-up of newcomers at postgraduate level academic years 1999/2000 and 2000/01] Högskoleverket, Sverige [Swedish National Agency for Higher Education]. https://www.uka.se/download/18.12f25798156a345894e2b8e/1487841900934/1201R-doktorander-forskarutbildning.pdf. Accessed 28 June 2019.

[11] Dahlin M, Fjell J, Runesson B (2010). Factors at medical school and work related to exhaustion ampng physicians in their first postgraduate year. Nordic journal of psychiatry.

[12] Pyhältö Kirsi, Toom Auli, Stubb Jenni, Lonka Kirsti (2012). Challenges of Becoming a Scholar: A Study of Doctoral Students' Problems and Well-Being. ISRN Education.

[13] Larsson C, Hensing G, Allebeck P (2003). Sexual and gender-related harassment in medical education and research training: results form a Swedish survey. Med Educ.

[14] Babaria P, Abedin S, Berg D, Nunez-Smith M (2012). I’m too used to it: a longitudinal qualitative study of third year female medical students’ experiences of gendered encounters in medical education. Soc Sci Med.

[15] Witte FM, Stratton TD, Nora LM (2006). Stories from the field: students’ descriptions of gender discrimination and sexual harassment during medical school. Acad Med.

[16] Stratton TD, McLaughlin MA, Witte FM, Nora LM (2005). Does students’ exposure to gender discrimination and sexual harassment in medical school affect specialty choice and residency program selection?. Acad Med.

[17] Noy C (2008). Sampling knowledge: the hermeneutics of snowball sampling in qualitative research. Int J Soc Res Methodol.

[18] COREQ guidelines for reporting qualitative studies. https://academic.oup.com/intqhc/article/19/6/349/1791966. Accessed 28 June 2019.

[19] Kvale S, Brinkman S (2009). Den kvalitaitva forskningsintervjun. [the Qualitiative research interview].

[20] Malterud K (1993). Shared understanding of the qualitative research process. Guidelines for the medical researcher. Fam Pract.

[21] Malterud K (2001). Qualitative research: standards, challenges, and guidelines. Lancet.

[22] Malterud K (2012). Systematic text condensation: a strategy for qualitative analysis. Scand J Public Health.

[23] Dahlgren E, Winkvist A. Qualitative Methodology for International Public Health. Epidemiology and Public Health Sciences, Department of Public Health and Clinical Medicine, Umeå University, 2007. http://explore.bl.uk/primo_library/libweb/action/search.do?fn=search&ct=search&vid=BLVU1&vl(freeText0)=9172643269. Accessed 28 June 2019. ISBN 978-91-7264-326-0.

[24] Golde CM (2000). Should I stay or should I go? Student descriptions of the doctoral attrition process. Rev High Educ.

[25] Wright T (2003). Postgraduate research students: people in context?. Br J Guid Couns.

[26] Lee NJ (2009). Professional doctorate supervision: exploring student and supervisor experiences. Nurse Educ Today.

[27] Lee A (2008). How are doctoral students supervised? Concepts of doctoral research supervision. Stud High Educ.

[28] Goldblatt H, Karnieli-Miller O, Neumann M (2011). Sharing qualitative research findings with participants: study experiences of methodological and ethical dilemmas. Patient Educ Couns.

[29] Bazrafkan L (2016). Management of stress and anxiety among PhD students during thesis writing: a qualitative study. Health Care Manag.

[30] Jeffe DB, Andriole DA, Wathington HD, Tai RH (2014). Educational outcomes for students enrolled in MD-PhD programs at medical school matriculation, 1995-2000: a national cohort study. Acad Med.

[31] Dijkhuizen K (2018). Encouraging residents’ professional development and career planning: the role of a development-oriented performance assessment. BMC Med Educ.

[32] Watts HJ (2010). Team supervision of the doctorate: managing roles, relationships and contradictions. Teach High Educ.

