# Supplementary material for: Doctoral students’ perceived working environment, obstacles and opportunities at a Swedish medical faculty: a qualitative study
Source: BMC Med Educ. 2019 Jul 8;19:250. doi: 10.1186/s12909-019-1684-x (PMC6615109; doi:10.1186/s12909-019-1684-x)
Supplement: Supplementary file 1 — The Interview guide. COREQ guidelines [18]. (PDF 147 kb) [file 12909_2019_1684_MOESM1_ESM.pdf]

Interview Guide

Open questions. Semi-structured.

1. What´s your subject for doctoral studies, allocation of time for dissertation, employment, occupation.

2. In general, what is your experience of doctoral studies?

3. What has been positive?

4. Is there anything that been less positive?

5. Have you ever been relegated because of your gender? Please describe the circumstances. Has it happened more than once? How did it affect you in that case?

6. Have you been aware that someone in your circle has been relegated because of their gender?

7. What do you think about the workload? Is it reasonable?

8. In the survey, there was a question on how to estimate your work ability on a scale from 0 to 10. Do you remember what you replied? What would your answer be now?

9. To what extent do you research in standby time and/or on holidays? What's the reason for that? How do you experience it?

10. To what extent do you get research time when you wish? If you are denied research time, will you be offered research time within periods you have not wanted, or are you denied research time altogether?

11. How is it/ has it been to combine doctoral studies with work and/or leisure?

12. Is there anything you want to add?
